# The Hippo/MST Pathway Member SAV1 Plays a Suppressive Role in Development of the Prehierarchical Follicles in Hen Ovary

**DOI:** 10.1371/journal.pone.0160896

**Published:** 2016-08-09

**Authors:** Zhichao Lyu, Ning Qin, Thobela Louis Tyasi, Hongyan Zhu, Dehui Liu, Shuguo Yuan, Rifu Xu

**Affiliations:** 1 Department of Animal Genetics, Breeding and Reproduction, College of Animal Science and Technology, Jilin Agricultural University, Changchun 130118, Jilin, China; 2 Jilin Grain Group Agriculture and Livestock Co., Ltd., Changchun 130062, Jilin, China; China Agricultural University, CHINA

## Abstract

The Hippo/MST signaling pathway is a critical player in controlling cell proliferation, self-renewal, differentiation, and apoptosis of most tissues and organs in diverse species. Previous studies have shown that Salvador homolog 1 (SAV1), a scaffolding protein which functions in the signaling system is expressed in mammalian ovaries and play a vital role in governing the follicle development. But the exact biological effects of chicken SAV1 in prehierarchical follicle development remain poorly understood. In the present study, we demonstrated that the SAV1 protein is predominantly expressed in the oocytes and undifferentiated granulosa cells in the various sized prehierarchical follicles of hen ovary, and the endogenous expression level of *SAV1* mRNA appears down-regulated from the primordial follicles to the largest preovulatory follicles (F2-F1) by immunohistochemistry and real-time RT-PCR, respectively. Moreover, we found the intracellular SAV1 physically interacts with each of the pathway members, including STK4/MST1, STK3/MST2, LATS1 and MOB2 using western blotting. And SAV1 significantly promotes the phosphorylation of LATS1 induced by the kinase of STK4 or STK3 in vitro. Furthermore, SAV1 knockdown by small interfering RNA (siRNA) significantly increased proliferation of granulosa cells from the prehierarchical follicles (6–8 mm in diameter) by BrdU-incorporation assay, in which the expression levels of *GDF9*, *StAR* and *FSHR* mRNA was notably enhanced. Meanwhile, these findings were consolidated by the data of SAV1 overexpression. Taken together, the present results revealed that SAV1 can inhibit proliferation of the granulosa cells whereby the expression levels of *GDF9*, *StAR* and *FSHR* mRNA were negatively regulated. Accordingly, SAV1, as a member of the hippo/MST signaling pathway plays a suppressive role in ovarian follicle development by promoting phosphorylation and activity of the downstream LATS1, may consequently lead to prevention of the follicle selection during ovary development.

## Introduction

Ovarian follicular development in chicken is an intricate and highly coordinated process involving a number of divergent biological effects on the maturation of oocytes, differentiation and proliferation of granulosa and theca cells within the follicles directed by multiple endocrine, paracrine, and autocrine regulatory factors [1–3]. In which, a wide variety of local intra-ovarian factors, such as steroidogenic acute regulatory protein (StAR), growth differentiation factor-9 (GDF9) and cyclin D2 (CCND2), were implicated in folliculogenesis, growth and development of the ovarian follicles as well as various members of the glycoprotein hormone family of gonadotropins, such as follicle-stimulating hormone (FSH) and FSH receptor (FSHR) [4–7]. And immediately before and after dominant follicle selection, the relatively higher expression levels of *FSHR* mRNA and protein are required and maintained within the granulosa cells of hen ovarian prehierarchical follicles [8]. Furthermore, many cell signaling systems were also involved in the developmental process, wherein the Hippo/MST signaling pathway was one of the most attractive research topics in recent years [9, 10]. The Hippo/MST signaling pathway has initially been identified in *Drosophila* as an essential regulator of cell proliferation and apoptosis during development [11, 12]. In mammals, major components of the pathway include the two upstream serine/threonine (Ser/Thr) kinases MST1 (mammalian Sterile 20-like kinase 1, a homologue of Hippo in *Drosophila*) and MST2, a scaffolding protein *Salvador* homolog 1 (SAV1 or WW45), two Ser/Thr protein kinase LATS1 (large tumor suppressor homolog 1) and LATS2 that interact with Mob1 protein and one transcriptional co-activator YAP1 (Yes-associated protein, *yorkie*) [7, 13, 14, 15]. However, the member of MST1/2 and Mob1 activates the LATS1/2 by multi-site phosphorylation [16, 17], and the activated LATS1/2 then targets the proto-oncogene YAP1 to promote their cytoplasmic localization or to bind with transcriptional enhancer activation domain family member (TEAD) for mediating YAP-dependent gene expression [15, 18]. In *Drosophila*, Hippo is the central component of an anti-proliferative pathway that responds to signals arising from cell-cell contact to regulate negatively the oncogenic transcriptional coactivator, *yorkie*. Loss of Hippo function results in a yorkie-dependent accelerated proliferation, resistance to apoptosis and massive organ overgrowth [19, 20, 21]. Furthermore, the Hippo signaling pathway has been identified to be a pivotal player in the regulation of stem cell proliferation, differentiation, migration and maturation in human [22, 23, 24], but the exact functions and regulatory mechanisms of Hippo signaling in the different tissues and organs exhibit shared or/and divergent characteristics [9, 15, 21, 25]. Moreover, the recent studies in Drosophila and mammals have also shown that the pathway plays a critical role in controlling maturation of oocytes, proliferation of granulosa cells, follicular atresia in folliculogenesis and ovarian follicle growth [9, 10, 26, 27]. Whether the Hippo/MST pathway in chicken comprises the same members as in mammals and *Drosophila*, and whether these members exert the similar or different roles in regulating the follicular development of ovary, however, has received little attention.

Our current understanding of the pathway in chicken is focused on *SAV1* gene, encoding protein SAV1 known to be a tumor suppressor in mammals and *Drosophila*, acts as a core partner of the Ser/Thr protein kinase MSTs (MST1 and MST2) [28, 29]. Previous studies have demonstrated that SAV1 recruits LATS to the MST to promote the phosphorylation of LATS mediated by MST1 (homologue of Ser/Thr kinase 4, STK4 in chicken) and MST2 (STK3 in chicken) [30, 31], and that SAV1 is required for the correct cellular localization and function of MST [32]. Moreover, SAV1 has been reported to have several phosphorylation residues that were phosphorylated by MST in human [33, 34]; the phosphorylation of SAV1 by MSTs promotes cell death [35]. Structural analysis has identified that SAV1 having the domains permits protein-protein interaction, including 2 WW domains and a coiled-coil motif in its C-terminus, which suggest that SAV1 functions as a scaffold in a multimeric complex [33]. Disruption of SAV1 in mice results in embryonic lethality with epithelial hyperplasia accompanied by defects in the terminal differentiation of various organs [32]. However, the functions and regulatory mechanism of the members of Hippo/MST pathway in follicular development have not been previously studied in hen ovaries, and furthermore, the detailed spatiotemporal localizations and biological effects of SAV1 on ovarian follicle development are poorly defined.

To explore the function and regulatory mechanism of Hippo/MST signaling pathway in ovarian follicle development, in the current work, we aimed to investigate the spatiotemporal expression changes of SAV1 in the various-sized developing follicles, verify the physical interaction of SAV1 with the core components (such as STK4/MST1, STK3/MST2 andLATS1) of the pathway, and further to reveal the biological roles of SAV1 protein in development of the prehierarchical follicles and its molecular regulation in hen ovary.

## Materials and Methods

### Animals and sampling

Birds sampled in this work were layers of Lohmann brown commercial line that were reared in laying batteries according to the management reported by us [36]; all birds for this experiment were obtained from the population and sacrificed at 21 weeks of age. Follicles in various sizes were taken from the hen ovaries according to the method of Stepińska and Olszańska (1996) [37]. A representative portion of each ovary was sampled and immediately frozen in liquid nitrogen, and stored at -80°C; and another equal part of the tissue was fixed using 4% neutral-buffered formalin at 4°C. All procedures performed in animals were approved by the Institutional Animal Care and Use Committee of Jilin Agricultural University (Changchun, China).

### Cell lines and cell culture

Chinese hamster ovary (CHO) cells were purchased from Shanghai Institute Cell Biology of Chinese Academy of Sciences (Shanghai, China) and preserved in our laboratory. Culture of granulosa cells (GCs) from hen prehierarchical follicles was performed according to the published method [38]. The cultured granulosa cells used in this experiment have been purified and quantified. The specificity of the granulosa cells been identified by the H & E staining procedure and fluorescence staining analysis [39].

### Immunohistochemistry

Tissue slides of the ovarian prehierarchical follicles were prepared, pretreated and blocked, and immunohistochemical staining was performed as our previously described procedures [39]. The slides were hybridized with rabbit anti-SAV1 (1:500, Sangon Co, Shanghai, China) as primary antibody incubated overnight at 4°C, and then incubated at room temperature for 1 h with goat anti-rabbit secondary antibody (1:1000) labeled with horseradish peroxidase (HRP). Photomicrographs of the sections were taken using a JNOEC XS-213 biological microscope (Jiangnan Optics & Electronics Co., Ltd. Nanjing, China).

### Quantitative real-time RT-PCR

To assess mRNA expression of target genes in the GCs, real-time quantitative reverse transcriptase PCR (qRT-PCR) was conducted according to our previously described method [39]. The primers used for *SAV1* gene: forward 5’-ATGAGGCGTGAAAGCAACAG-3’ and reverse 5’-CCGCTGTGCTCATAGTATCTGTA-3’. The *18S rRNA* gene was used as an inner control in each reaction system: forward 5’-TAGTTGGTGGAGCGATTTGTCT-3’ and reverse 5’-CGGACATCTAAGGGCATCACA-3’. The other primers utilized for amplification of the *FSHR*, *GDF*9, *STAR* and *CCND2* genes were listed in Table 1. Using the 2^-ΔΔCt^ method, mRNA expression results were normalized against *18S rRNA* as internal control.

**Table 1:** Primer pairs designed for quantitative real-time PCR.

Gene	Forward primer (5′—3′)	Reverse primer (5′—3′)	Accession No.	Size
*SAV1*	ATGAGGCGTGAAAGCAACAG	CCGCTGTGCTCATAGTATCTGTA	XM_015276749.1	236 bp
*FSHR*	AATACCCTGCTAGGACTG	GAATACCCATTGGCTCA	NM_205079.1	238 bp
*GDF9*	ACTTTCACTCGGTGGATT	ATGCTGGGACATACTTGG	AY566700.2	175 bp
*STAR*	GCCAAAGACCATCATCAAC	TCCCTACTGTTAGCCCTGA	NM_204686.2	141 bp
*CCND2*	AACTTGCTCTACGACGACC	TTCACAGACCTCCAACATC	NM_204213.1	150 bp
*18SrRNA*	TAGTTGGTGGAGCGATTTGTCT	CGGACATCTAAGGGCATCACA	AF173612.1	169 bp

### Construction of recombinant plasmids

Chicken *SAV1* cDNA sequence (GenBank accession: XM_015276749.1) was amplified from a chicken cDNA library by PCR and subcloned into a pFLAG-CMV-2 expression vector (Sigma, St. Louis, MO, USA) to generate pFLAG-SAV1 expression construct (S1 Table). Similarly, the *SAV1* cDNA sequence was also subcloned into a pSF-CMV-Puro-NH2-GST expression plasmid (Sigma, St. Louis, MO, USA). The structure of FLAG-fusion or GST-fusion plasmids was confirmed by sequencing with BigDye v.3.1 (ABI Applied Biosystems, Sangon Co, Shanghai, China). The cDNA sequences of chicken *STK4*, *STK3*, *LATS1* and *MOB2* open reading frames were amplified by PCR using the full-length *STK4* cDNA (NM_001030853.1), *SKT3* (NM_001031337.2), *LATS1* (XM_419666.3) and *MOB2* (XM_004941451.1) as template and then subcloned into a pcDNA3.0 expression vector (Invitrogen, Carlsbad, CA, USA), respectively. Similarly, the cDNA sequences of *LATS1* gene was subcloned into a pCMV-HA-N expression vector (Clontech, Mountain View, CA, USA). By this way the recombinant expression constructs pcDNA3.0-STK4, pcDNA3.0-STK3, pcDNA3.0-LATS1, pCMV-HA-LATS1 and pcDNA3.0-MOB2 were created. Details of the plasmid constructions were listed in (S1 Table).

### Cell transfection

Transfection for *SAV1* gene expression by using the recombinant plasmid vector pFLAG-SAV1 was performed as reported previously [7]. Briefly, the granulosa cells grouped randomly were transfected by the plasmid pFLAG-SAV1 and pFLAG blank vector using Lipofectamine 2000 transfection reagent (Invitrogen, USA). Culture (1×10^5^ cells/well in a 24-well plate) were conducted in a basal medium containing 1μl/ml Polybrene (hexadimethrine bromide, Sigma), and incubated at 37 °C with 5% CO_2_. After being continually cultured for 24 h, the granulosa cells were collected and then lysed for immunoblot analysis and qRT-PCR analysis.

For in vitro protein binding studies using Glutathione S-transferase (GST) pull-down method, CHO cells were transfected with recombinant pSF-GST-SAV1 construct or pSF-GST empty vector, and the CHO cells were co-transfected with the expression constructs of pcDNA3.0-STK4, STK3, LATS1 and MOB2, or the empty pcDNA3.0 vector based upon the method [7, 40], respectively. For an experiment of induced LATS1 phosphorylation assay, CHO cells were also transfected with pCMV-HA-LATS1 expression reconstruct or vacant pCMV-HA expression vector.

### Western blotting

Following cell transfection test, western blot analysis for SAV1, phospho-SAV1, STK4, STK3, LATS1, phospho-LATS1, MOB2 and YAP1 protein was conducted as previously described [39] using total cellular extracts. Briefly, equivalent amounts of protein were separated by 10% (w/v) SDS-polyacrylamide gel under reducing conditions and electro-transferred to Protran nitrocellulose membranes (Whatman, Dassel, Germany). The affinity purified antibodies for SAV1 and the others were used **(**S2 Table**)**. The horseradish peroxidase-conjugated anti-rabbit or anti-mouse IgG secondary antibody was incubated for 2 h at room temperature. Blots were subsequently performed with ECL western blotting agent (Rockford, IL, USA) for 5 min and exposed to X-ray film for 1–5 min. The outcome was visualized by the ECL Plus Western blotting detection system according to the manufacturer's instructions. Anti-β-actin (dilution 1:1000, Boster, China) antibody acted as loading control.

### Immunoprecipitation assay

As stated above, the GCs were transfected with the pFLAG-SAV1 expression construct for 24 h in normal culture media and then lysed and immunoprecipitated in an buffer containing 50 mM Tris—HCl, pH 7.4, 150 mM NaCl, 0.2 mM PMSF, 1% Triton X-100 and 1 mM EDTA, as described previously [41]. For negative controls, the cell lysates were immunoprecipitated and incubated with chicken IgG (Sangon Co, Shanghai, China). The eluted samples or cell lysates were added to 4× SDS sample buffer and heated at 95°C for 5 min to denature the proteins. Samples were then subjected to SDS-PAGE for western blotting.

### GST pull-down assay

After the CHO cells were co-transfected with pSF-GST-SAV1 and each of the plasmids of pcDNA3.0-STK4, STK3, LATS1 and MOB2 for 24 h, respectively; the cells were lysed and coimmunoprecipitated as described [40] with a little modification. Briefly, the cell lysates were incubated with Glutathione Sepharose 4B beads (Beyotime Institute of Biotechnology) overnight. The beads were collected by centrifugation at 3,000 rpm for 3 min at 4°C, washed three times with lysis buffer. Following boiling with 1× SDS-PAGE protein loading buffer for 5 min, 20 μl of the supernatant was separated by SDS-PAGE and transferred to a PVDF membrane. After blocking with 5% skimmed milk at 4°C, the membrane was incubated with primary antibody and secondary antibody (HRP-goat anti-rabbit or anti-mouse IgG) list in S2 Table. The co-transfection of CHO cells with empty pSF-GST vector and the pcDNA3.0-STK4, STK3, LATS1 and MOB2 for 24 h was used as a negative control. Co-immunoprecipitated proteins were detected by western blotting and autoradiography.

### In vitro phosphorylation assay

CHO cells were transfected with the pCMV-HA-LATS1 expression construct or an empty pCMV-HA vector for 24 h and then lysed, cell lysates were immunoprecipitated using an antibody to HA. The phosphorylation assay was conducted based upon the reported procedures [34] with a bit modification Briefly, following the immunoprecipitates were resolved by 10% SDS-PAGE and analyzed by western blotting with the LATS1 antibody, equivalent amounts of the purified LATS1 protein sample were added to the lysis supernatants from cells transfected with the pFLAG-SAV1 construct or an empty pFLAG-CMV-2 vector alone, or co-transfected with the the pcDNA3-STK4 or STK3 expression vector as aforementioned (S1 Table), Blots were subsequently performed with ECL western blotting agent (Rockford, IL, USA) using the primary and secondary antibodies (S2 Table).

### Transfection of siRNA

Specific siRNAs targeting *SAV1* gene were designed using an Invitrogen siRNA Wizard v3.1 [42]. All designed siRNA sequences were blasted against the chicken genome database to eliminate cross-silence phenomenon with nontarget genes. A most effective SAV1 specific siRNA was further screened by qRT-PCR and Western blotting: 5′- GCUUGCAUGAGGACUACAGAU -3′. Scrambled siRNA that does not target any gene was used as the negative control siRNA: 5′- GUAUGACGAUCCGACCUAGUCTT -3′. As mentioned above, GCs were plated in 24-well plates and the siRNAs were transfected into the culture cells with Lipofectamine 2000 (Invitrogen, USA) according to the manufacturer's instructions.

### Cell proliferation assay

Following the procedure of cell transfection for SAV1 expression, variation of cell proliferation was detected using BrdU (5'-bromo-2'-deoxyuridine) Cell Proliferation Assay Kit (CST Biological Reagents Co. Shanghai, China) according to the manufacturer’s protocol. Briefly, the control and transfected cells were seeded at a density of 1×10^4^ cells/well in 96-well flat-bottom and cultured for 48 h. The proliferation assay was performed 12 h following the addition of BrdU reagent (10 ng/ml). The absorbance values measured at 450 nm wavelength represented the rate of DNA synthesis and corresponded to the number of proliferating cells. Each experiment was performed in triplicate and repeated five times. The number of BrdU^+^ cells was expressed as percentage and calculated relative to the total number of cells counted in the microscope fields observed in the negative control. Twenty fields were analyzed and averaged per each condition to give the final data shown.

### Statistical analysis

Statistical calculation was performed using the SPSS12.0 software package [43]. All the experiments were repeated at least three times using different batches of sampled birds. In quantifying mRNA expression levels by qRT-PCR analysis, four amplified products from independent reactions per bird were utilized. The data were collected and analyzed with a one-way ANOVA and Tukey’s multiple-comparison test when more than two groups were involved, or using a Student’s *t* test when treatment and control groups were compared after confirmation of normal distributions for parametric analysis. *P <* 0.01 or *P <* 0.05 was accepted to be statistically significant.

## Results

### Expression of *SAV1* gene in prehierarchical follicles

**Fig 1:**
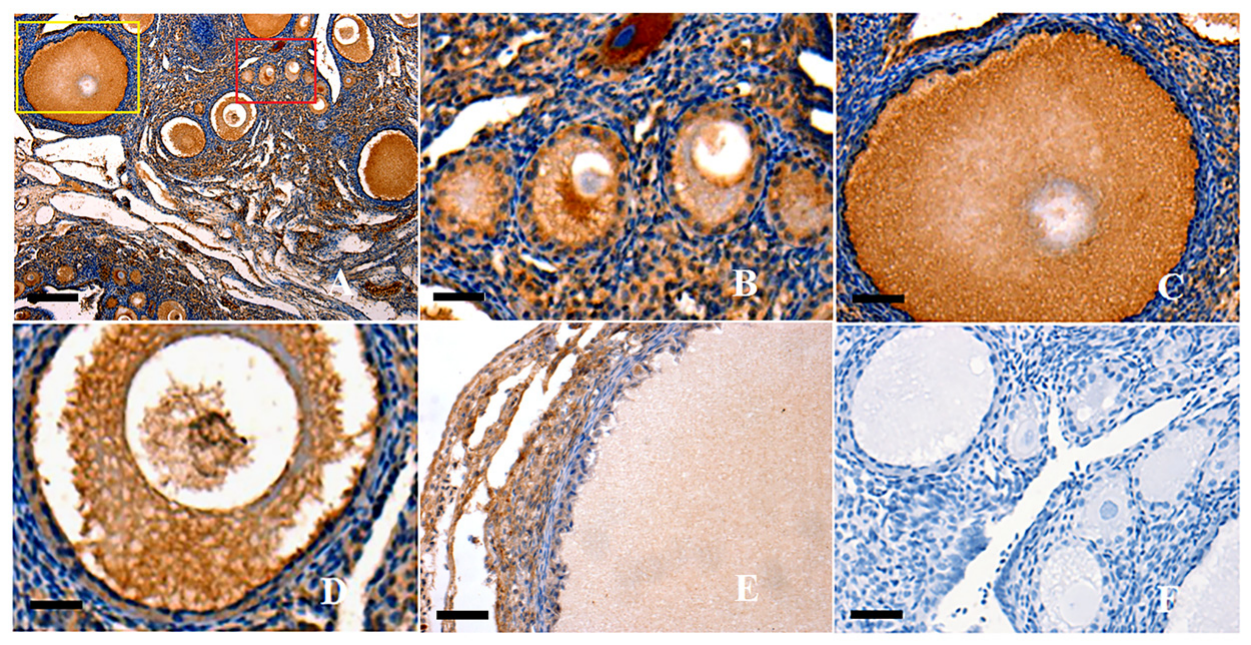
Immunohistochemical analysis of SAV1 protein expression in the ovarian follicles. Paraformaldehyde-fixed tissue sections were immunostained using anti-chicken SAV1 as described above. Panel A, strong staining is observed in all oocytes (OC) and granulosa cells (GCs) within the variously sized prehierarchical follicles (×10). Panel B, the amplified primordial follicle and primary follicle (×100), paralleling the one marked in the read box of panel A; Panel C, the amplified developing PF follicles with two or three layers of GCs (×20), paralleling the one marked in the yellow box of panel A. Panel D, the amplified small PF follicles with one or more layers of GCs (×40). Panel E, the amplified large PF follicles with more layers of GCs (×20). Panel F, one of the negative controls for SAV1, the sections were immunostained with pre-immune serum, no significant expression was detected. Negative control sections were prepared with the primary antibody pre-incubated with a blocking peptide for 1 h at room temperature and for 2 h at 4°C. Five birds were used for immunohistochemical analysis and representative microscopic fields were selected. Scale bar = 100 μm.

This study initially localized the SAV1 protein in variously sized prehierarchical follicles (PF) using immunohistochemistry. It was indicated that SAV1 protein was predominantly expressed in the oocytes and undifferentiated GCs from primordial follicles (less than 20μm in diameter), primary follicles (30–90 μm in diameter) and PF (60 μm to 8 mm in diameter) examined; but an undetectable staining was observed in the theca cells of the follicles (Fig 1). Strong immunostainings were observed in the primordial follicles, primary and small sized PF follicles (<1 mm in diameter) (Fig 1A–1C). As follicular development, the positive staining was becoming weaker in the oocytes and granulosa cells from the large follicles than that from the smaller ones (Fig 1D and 1E). Furthermore, SAV1 protein was also found to be expressed in ovarian stroma (mainly containing stromal cells, reticular and collagen fibers) that extensively dispersed among all of the examined follicles at the various development stages.

**Fig 2:**
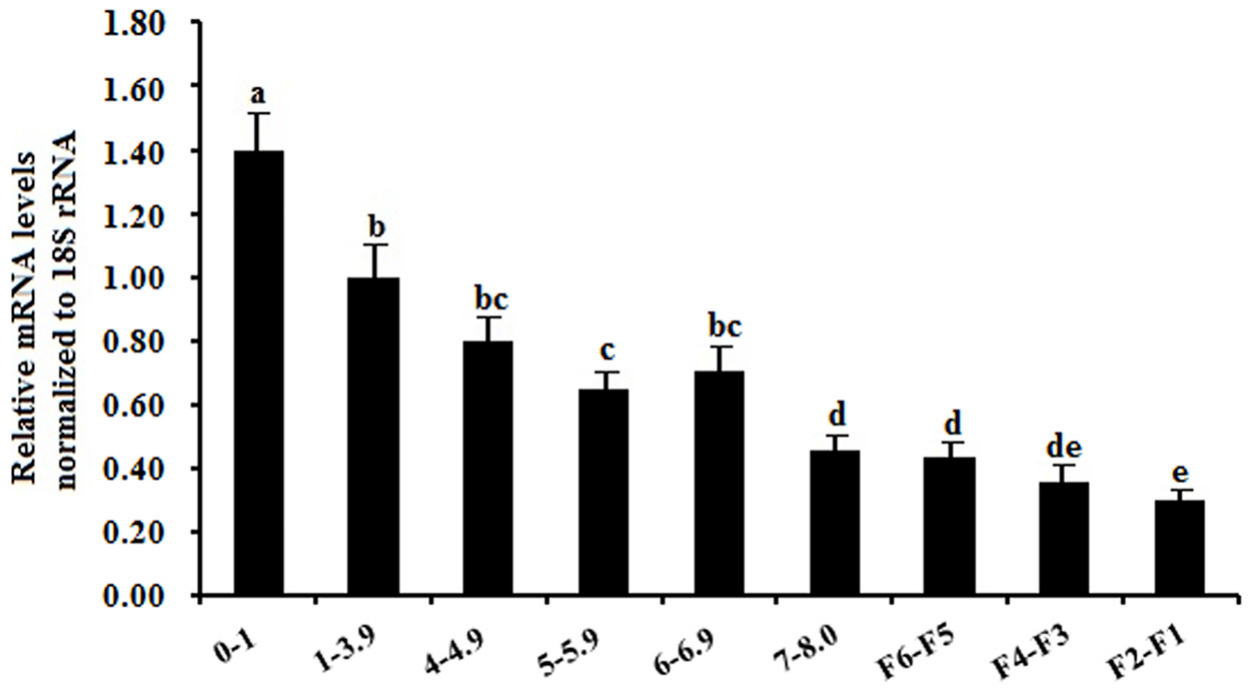
Quantification of expression levels of the *SAV1* mRNA in variously sized follicles from freshly collected ovaries. The data are the mean ± SEM from 10 hens (n = 10), and bars with different letters above them differ significantly in the amount of mRNA expression as normalized to the *18S rRNA* gene (*P* < 0.05). Horizontal ordinate represents the examined follicle sizes as indicated: 0-1mm, small follicles (<1 mm in diameter); 1–3.9 mm, the prehierarchical follicles were from 1 to 3.9 mm in diameter; 4–4.9 mm, from 4 to 4.9 mm in diameter; and the others followed similarly.

As development of the ovarian follicles, expression levels of *SAV1* mRNA showed a significant difference within the developmental stages of the follicles. Wherein, a highest expression level of *SAV1* mRNA abundance was determined at the smallest follicles sampled (<1 mm in diameter), and the endogenous expression level of *SAV1* mRNA appears down-regulated from the smallest prehierarchical follicles to the largest preovulatory follicles (F2-F1) in the chicken ovary (Fig 2).

### Interaction between SAV1 and the members of Hippo pathway by coimmunoprecipitation

**Fig 3:**
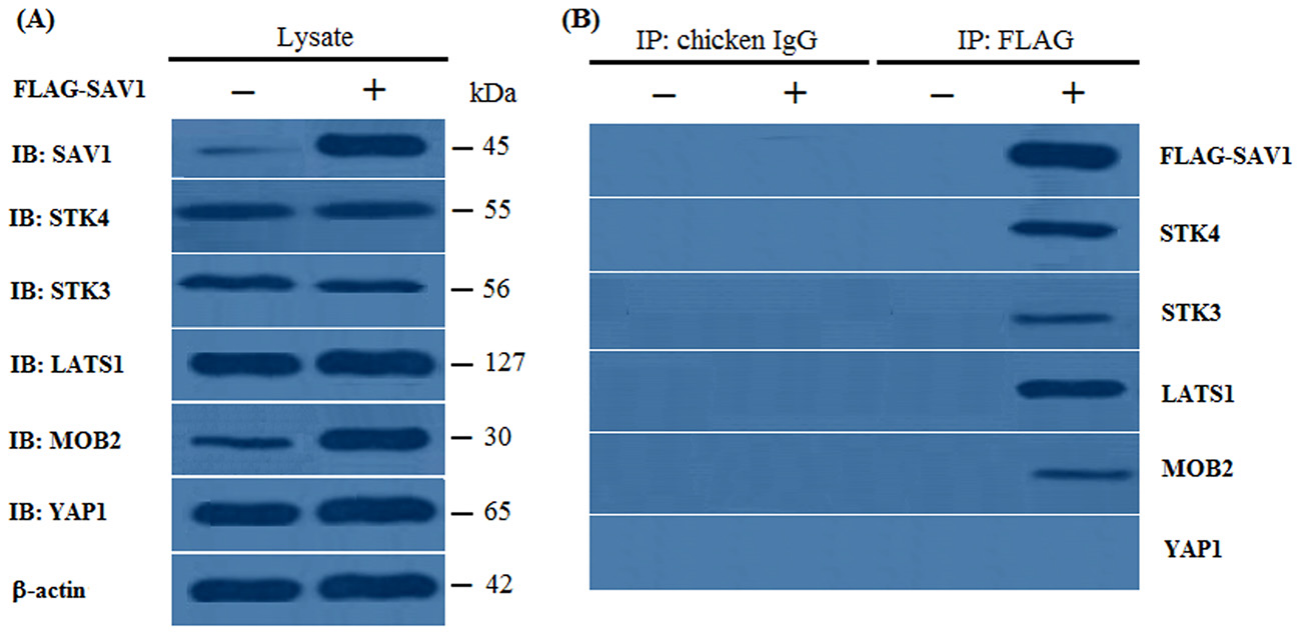
Interaction of SAV1 and STK4, STK3, LATS1, MOB2 and YAP1 by an coimmunoprecipitation experiment. Chicken GC cells were transfected with an empty pFLAG-CMV-2 expression vector (−) or pFLAG-SAV1 expression construct (+). After transfection for 24 h, the cells were lysed, and the lysates were immunoprecipitated with a control chicken IgG or an antibody to FLAG. The lysates (Lysate) and immunoprecipitates (IP) were analyzed by immunoblotting (IB) with SAV1, STK4, STK3, LATS1, MOB2 and YAP1 antibodies. (A) When the empty pFLAG-CMV-2 vector was used as a template (Lysate, −), endogenous SAV1 was detected with a faint band observed, when the pFLAG-CMV-2-SAV1 construct was used as a template (Lysate, +), SAV1 was synthesized. The endogenous STK4, STK3, LATS1, MOB2 and YAP1 expression was also detected in the lysates prior to immunoprecipitation. The β-actin was used as the loading control (B) When these lysates were immunoprecipitated with chicken IgG, no band was obtained for FLAG-SAV1in immunoprecipitates from cells expressing FLAG-SAV1, and no band was obtained for all of STK4, STK3, LATS1, MOB2 and YAP1(IP: chocken IgG). When the lysates were immunoprecipitated with an antibody to FLAG, endogenous STK4, STK3, LATS1 and MOB2 was coimmunoprecipitated in cells expressing FLAG-FOXL2 (IP: FLAG, +), but YAP1 was not coimmunoprecipitated in the cells. No one of the proteins was examined in the cells expressing the empty expression vector (IP: FLAG, −).

It was shown that when the pFLAG-CMV-2 vector was used as a template for protein synthesis, little endogenous SAV1 was detected with a faint band observed, but when the pFLAG-SAV1 vector was utilized as a template, SAV1 was largely synthesized in the GC lysate, with a strong band examined (Fig 3A). Meanwhile, some endogenous STK4, STK3, LATS1, MOB2 and YAP1 expression was also detected in the GC lysates (Fig 3A). When the lysates from these cells were immunoprecipitated with the control chicken IgG, no band was obtained for pFLAG-SAV1 in the coimmunoprecipitates from cells expressing FLAG-SAV1, or for control expressing the empty pFLAG-CMV-2 vector, and not even any band was obtained for SAV1, STK4, STK3, LATS1, MOB2 and YAP1 (IgG; Fig 3B). In contrast, when the same lysates were immunoprecipitated with an antibody to FLAG, both FLAG-SAV1 and each of SAV1, STK4, STK3, LATS1 and MOB2 (except for YAP1) were observed in the coimmunoprecipitates from cells expressing FLAG-SAV1, while not in coimmunoprecipitates from cells expressing the empty pFLAG-CMV-2 vector (FLAG; Fig 3B). This result indicated that the intracellular SAV1 physically interacts with each of the members including STK4, STK3, LATS1 and MOB2, in the GC cells of the prehierarchical follicles in hen.

### Interaction between SAV1 and the members of Hippo pathway by GST pull-down

**Fig 4:**
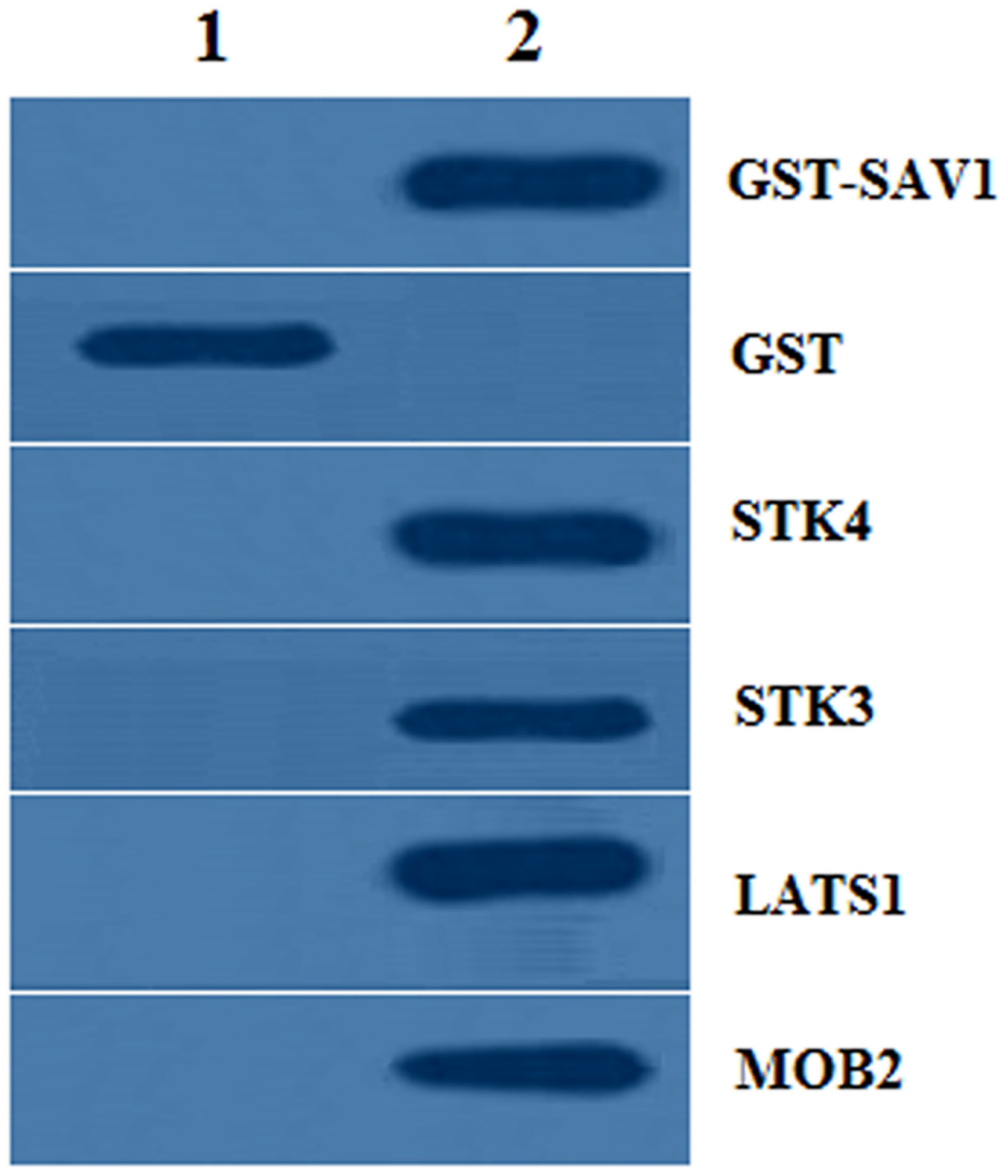
Western blotting analysis for GST pull-down samples. 1, Cell lysates from the CHO cells (which do not express endogenous chicken SAV1, STK4, STK3, LATS1 and MOB2) cotransfected by empty pSF-GST and each of the pcDNA3.0-STK4, pcDNA3.0-STK3, pcDNA3.0-LATS1 and pcDNA3.0-MOB2 expression constructs, respectively. 2, Cell lysates from GST-SAV1 and each of the pcDNA3.0-STK4, STK3, LATS1 and MOB2 transfected cells, respectively. The co-transfection of CHO cells with empty pSF-GST vector and the pcDNA3.0-STK4, STK3, LATS1 and MOB2 for 24 h was used as a negative control. GST, glutathione S-transferase

The GST pull-down detection demonstrated that the GST- SAV1 fusion protein could bind with STK4, STK3, LATS1 and MOB2, individually; but the GST-tagged proteins were not capable of binding in the CHO cells (Fig 4). Therefore, the present result further validated the interaction of SAV1 and each of the members of STK4, STK3, LATS1 and MOB2.

### Phosphorylation of SAV1 by SKT3 and SKT4

**Fig 5:**
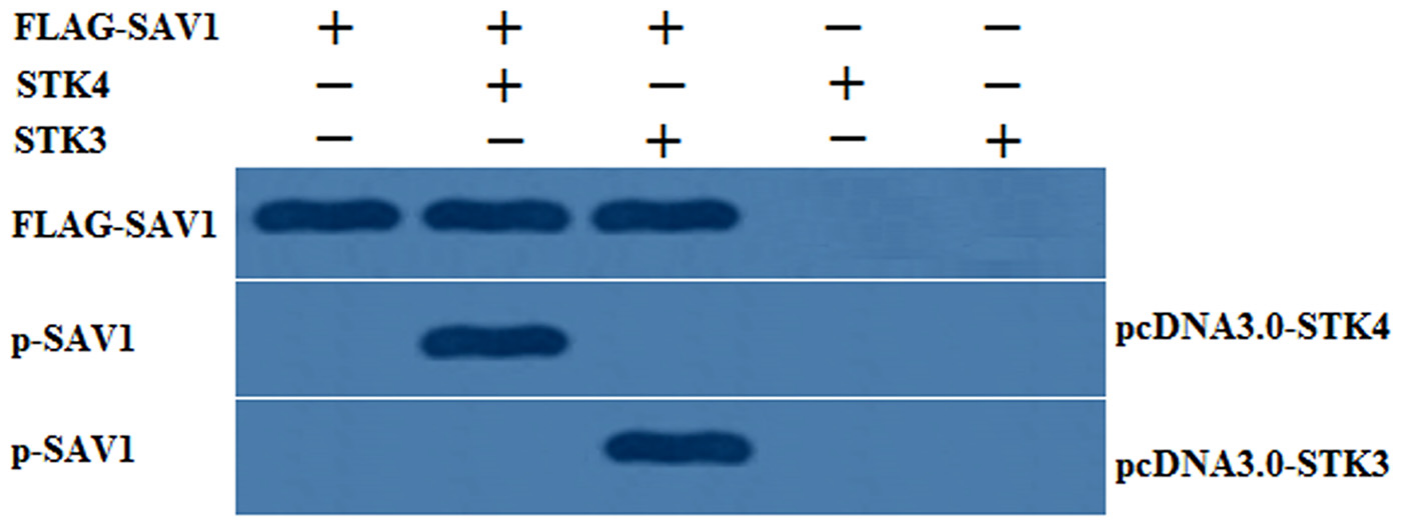
Phosphorylation of SAV1 by SKT3 and SKT4 in vitro. CHO cells (which do not express endogenous chicken SAV1, STK4 and STK3) was co-transfected with the pFLAG-SAV1 expression construct or an empty pFLAG-CMV-2 vector, and the pcDNA3-STK4 or STK3 expression vector or a vacant pcDNA3 vector for 24 h. The cells were lysated and analysed by immunoblotting procedure using an antibody to SAV1 and phosphorylated SAV1 (p-SAV1). The bands were observed corresponding to the antibody of p-SAV1 in the presence of STK4 or STK3 in the cell lysates, but no band was detected for the same lysates in the absence of STK4 or STK3.

As shown in Fig 5, when the FLAG-tagged SAV1 was co-expressed the presence of STK4 or STK3 in the cells, the lysates were immunoprecipitated corresponding to the antibody of p-SAV1, but no band was detected for the same lysates in the absence of STK4 or STK3. The present result confirmed that SAV1 was a phosphorylation substrate of SKT4 and SKT3 that may mediate the Hippo/MST pathway to control cell proliferation and follicle development.

### SAV1 promoting phosphorylation of LATS1

**Fig 6:**
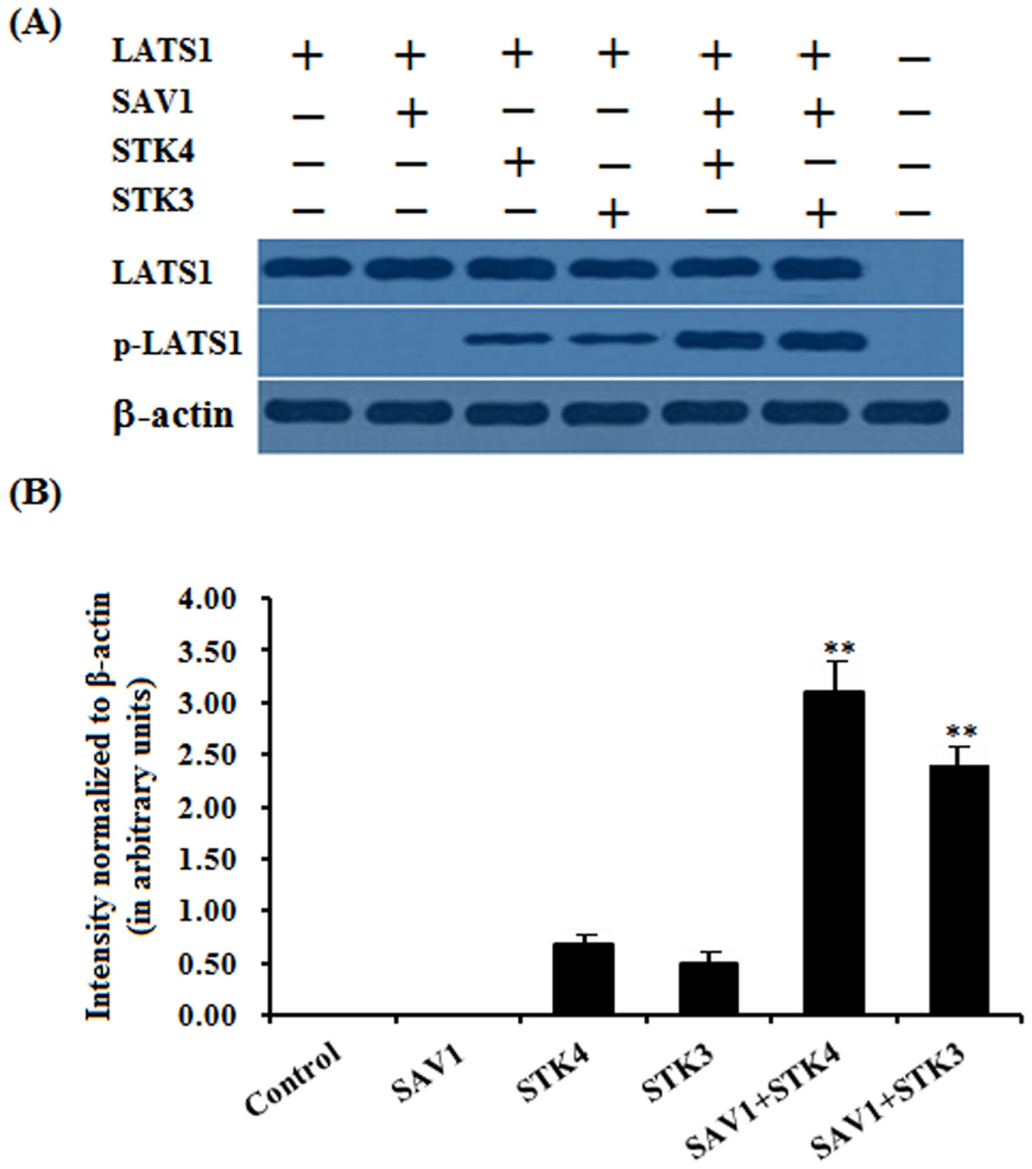
Phosphorylation of LATS1 by SAV1 in vitro. (A) CHO cells (which do not express endogenous chicken LATS1) was transfected with the pCMV-HA-LATS1 expression construct or an empty pCMV-HA vector for 24 h and lysed, cell lysates were immunoprecipitated using an antibody to HA. The immunoprecipitates were analyzed by western blotting with the LATS1 antibody, and the purified LATS1 protein was then used as a substrate to be incubated with the lysates from the cells transfected with the pFLAG-SAV1 construct or an empty pFLAG-CMV-2 vector alone, or co-transfected with the the pcDNA3-STK4 or STK3 expression vector aforementioned for an in vitro phosphorylation assay. The β-actin was used as the loading control. (B) Blotting signal intensity was quantified densitometrically after phosphorimaging (shown in A), and normalized for loading by comparison to the signal for β-actin. The signal intensity of LATS1 or phosphorylated LATS1 was expressed as the ratio β-actin signal in arbitrary units shown in B (n = 5 per mean ± SEM). The Five independent experiments were carried out in triplicate. The results are representative of at least three independent experiments. Statistical significance was marked with different superscript symbols ** P<0.01,* P<0.05.

To explore whether SAV1 induced phosphorylation of LATS1, as a physically interactive molecule with SAV1, CHO cells (which do not express endogenous chicken LATS1) was transfected with the pCMV-HA-LATS1 expression construct or an empty pCMV-HA vector and the cell lysates were immunoprecipitated using an antibody to HA. The immunoprecipitates were analyzed by western blotting with the LATS1 antibody, and the purified LATS1 protein was then used as a substrate to be incubated with the lysates from the cells transfected with the pFLAG-SAV1 construct or an empty pFLAG-CMV-2 vector alone, or co-transfected with the pcDNA3-STK4 or STK3 expression vector aforementioned for an in vitro phosphorylation assay. As shown in Fig 6, STK4 or STK3 was able to induced phosphorylation of LATS1 with a very faint band observed, but SAV1 was not capable of inducing phosphorylation of LATS1 (Fig 6A). However, a strong phosphorylated LATS1 band was detected in presence of both SAV1 and STK4, or SAV1 and STK4, respectively. These results indicated that SAV1 significantly promotes the phosphorylation of LATS1 induced by the kinase of STK4 or STK3 in vitro (Fig 6B).

### Effect of *SAV1* knockdown by RNA interference on granulosa cell proliferation

**Fig 7:**
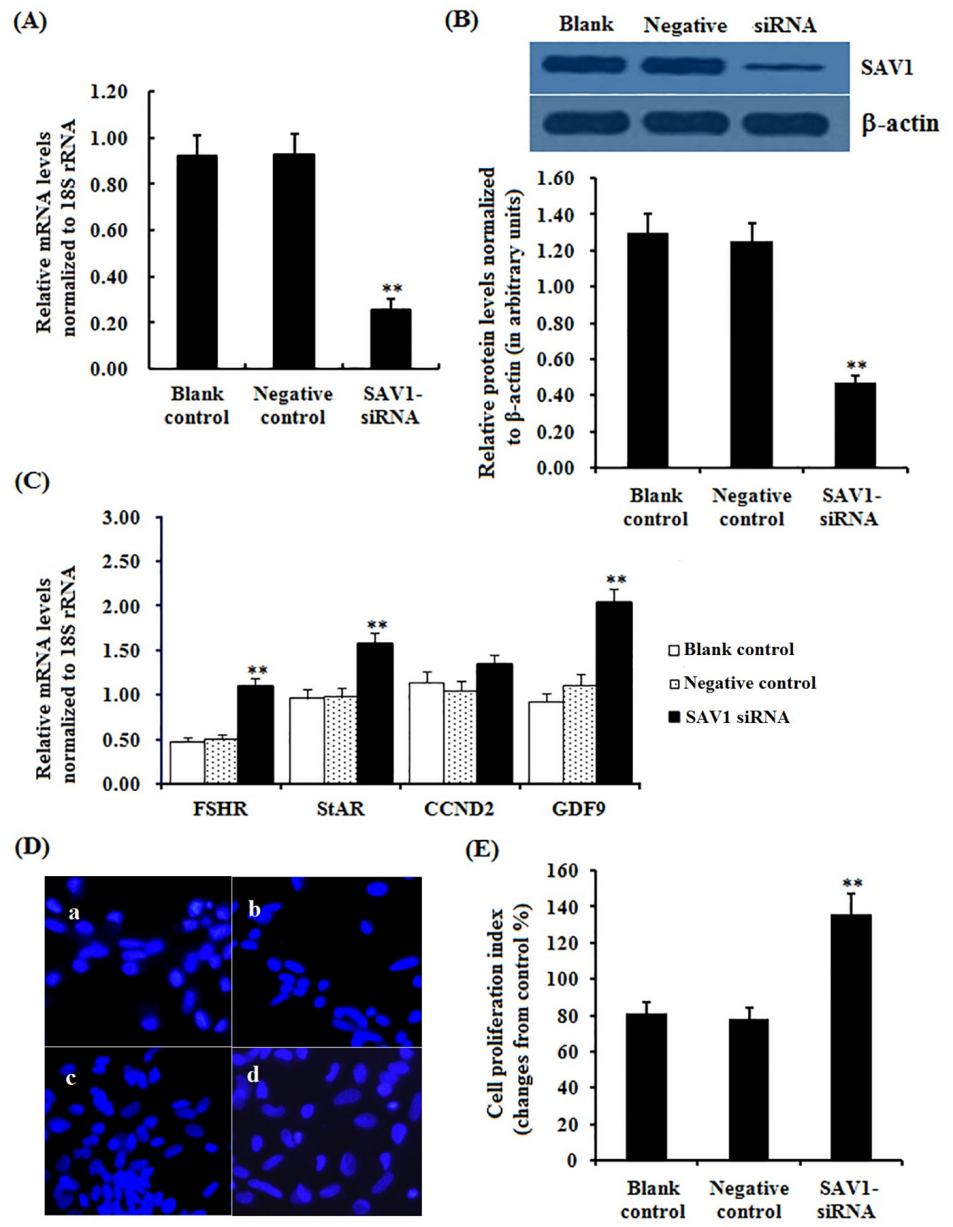
Effects of silencing *SAV1* on expression of *FSHR*, *StAR*, *CCND2* and *GDF9* mRNA examined and cell proliferation. Granulosa cells were transfected with specific siRNAs targeting *SAV1* gene, scrambled siRNA (negative control) and no siRNA (blank control). (A) The expression of *SAV1* gene before and after the GCs transfected with specific siRNA for 24 h was examined by qRT-PCR. Quantification of mRNA expression as normalized to the *18S rRNA* gene, the values on the bar graphs are the mean ± SEM of 10 hens (n = 10) from a representative experiment. (B) Expression levels of SAV1 protein in the GCs before and after the specific siRNA interference (RNAi) was detected by western blotting. The β-actin was used as the loading control. (C) The influence of silencing *SAV1* on *FSHR*, *StAR*, *CCND2* and *GDF9* mRNA expression in the granulosa cells from prehierarchichal follicles (6 to 8 mm in diameter) was examined. (D) The effects of silencing SAV1 on the GC proliferation were detected by BrdU incorporation assay (original magnification × 200). Of which panel a, blank control; panel b, negative control; panel c-d, RNAi group. (E) The number of BrdU^+^ cells was expressed as percentage and calculated relative to the total number of cells counted in the microscope fields observed in the negative control. For each group, bars with superscript symbol indicates that difference was significant compared to the control group ** P<0.01,* P<0.05.

As shown in Fig 7, in the cells transfected with SAV1-specific siRNA, the expression of SAV1 at the mRNA and protein levels decreased significantly (P<0.01). Expression levels of *FSHR*, *StAR* and *GDF9* mRNA were remarkably increased in the cells after transfected by the SAV1-specific siRNA (P<0.01), but no changes were detected in the *CCND2* mRNA expression levels before and after the siRNA transfections (P>0.05). Knock-downing *SAV1* expression, due to repressing effect of *SAV1* on the cell proliferation was attenuated, proliferation index of the GCs was sharply enhanced compared to the negative control (P<0.01).

### Effect of *SAV1* overexpression on granulosa cell proliferation

**Fig 8:**
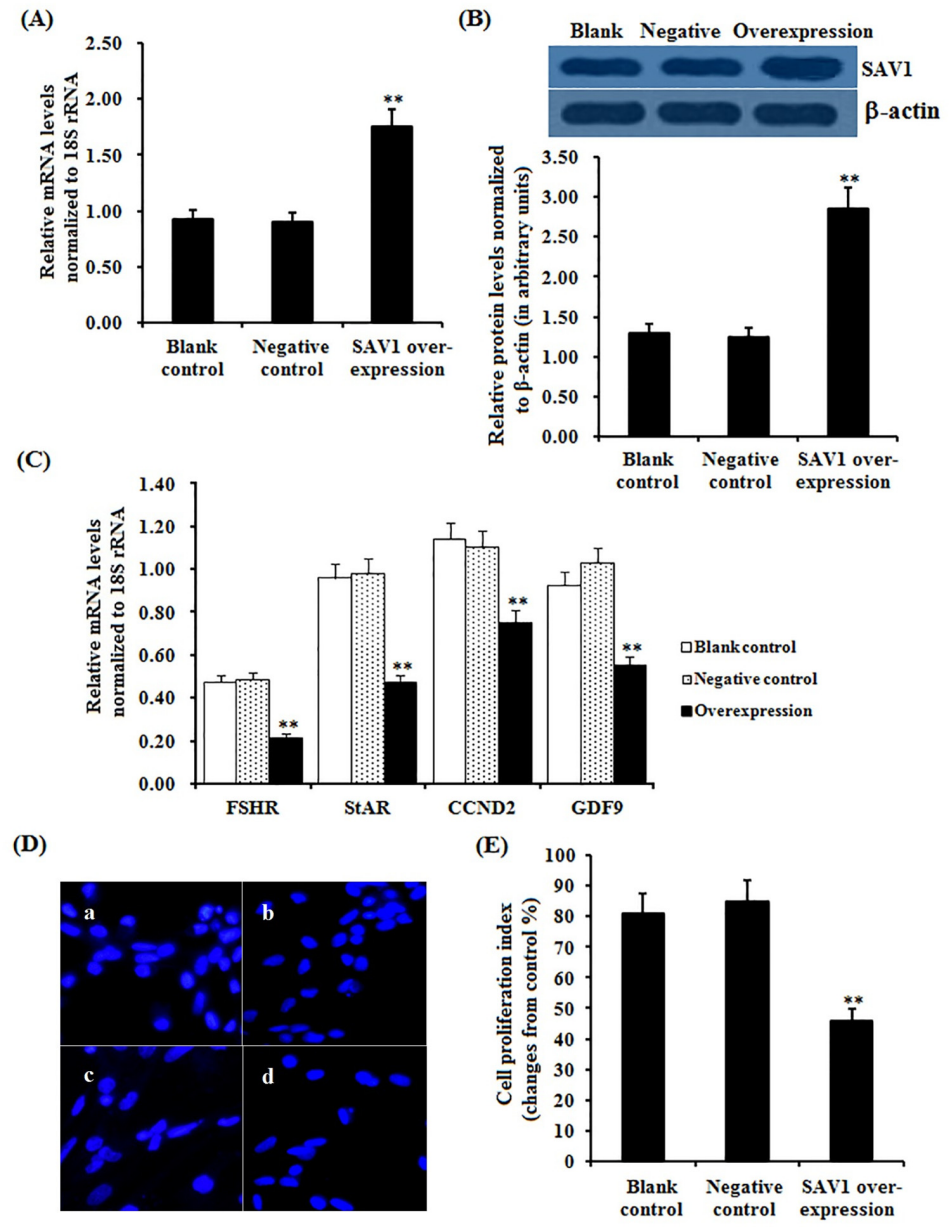
Effects of overexpressing *SAV1* on expression of *FSHR*, *StAR*, *CCND2* and *GDF9* mRNA examined and cell proliferation. Granulosa cells were transfected with reconstructed pFLAG-SAV1 plasmids, pFLAG CMV-2 empty vector (negative control) and no plasmid (blank control). (A) The expression of *SAV1* gene before and after the GCs transfected with pFLAG-SAV1expression vector for 24 h was examined by qRT-PCR. Quantification of mRNA expression as normalized to the *18S rRNA* gene, the values on the bar graphs are the mean ± SEM of 10 hens (n = 10) from a representative experiment. (B) Expression levels of SAV1 protein in the GCs before and after the transfection with with pFLAG-SAV1 vector was detected by western blotting. The β-actin was used as the loading control. (C) The influence of *SAV1* overexpression on *FSHR*, *StAR*, *CCND2* and *GDF9* mRNA abundances in the granulosa cells from prehierarchichal follicles (6 to 8 mm in diameter) was examined. (D) The effects of SAV1 overexpression on the GC proliferation were detected by BrdU incorporation assay (original magnification × 200). Of which panel a, blank control; panel b, negative control; panel c-d, RNAi group. (E) The number of BrdU^+^ cells was expressed as percentage and calculated relative to the total number of cells counted in the microscope fields observed in the negative control. For each group, bars with superscript symbol indicates that difference was significant compared to the control group ** P<0.01,* P<0.05.

To confirm the suppressive effect of SAV1 on GC proliferation, overexpression of *SAV1* gene in the GCs was performed by transfections with the pFLAG-SAV1 expression construct and pFLAG-CMV-2 blank vector, and a significantly increased level of mRNA and protein expression of SAV1 gene was detected in the GCs after transfected with the pFLAG-SAV1 expression vector (P < 0.01), as shown in Fig 8. In the cells transfected by the pFLAG-SAV1 plasmid, GCs proliferation index was significantly decreased compared to the negative control (P<0.01), furthermore, expression levels of *FSHR*, *StAR*, *CCND2* and *GDF9* mRNA in the GCs were notably down-regulated (P<0.01). These results consolidated that SAV1 protein plays a suppressive role in GC proliferation of the hen ovarian follicles.

## Discussion

The Hippo/MST signaling pathway has been identified as an important player in regulation of apoptosis and cell cycle control in Drosophila and mammals [12, 13, 15]. The Sav (SAV1 in chicken) is one of the most crucial members of the pathway that have been shown to restrict cell number by coordinating cell-cycle exit and apoptosis during Drosophila development [44]. In recent years, studies have been intensively focused on effects of the Hippo signaling on ovarian follicle development and growth, aging and tumorigenesis in human and mice [9, 27, 45], however, the spatiotemporal expression characters and the exact functions of chicken SAV1 in follicle development remains unknown. In the present study, we initially localized the SAV1 protein by using immunohistochemistry in an attempt to explore the possible role of SAV1 factor in regulation of the prehierarchical follicular development in laying hens. The specifically positive signals for SAV1 found in oocytes and GCs from primordial follicles and undifferentiated prehierarchical follicles 60 μm to 8 mm in diameter suggests its functional importance in oocyte maturation and in granular cell proliferation, and differentiation during the follicular growth. Meanwhile, these data also suggested that the intracellular SAV1 may be involved in the Hippo/MST pathway by an autocrine manner to predominate its regulative activity in the follicular development. Moreover, as development of the follicles in chicken, levels of *SAV1* mRNA expression showed a significant difference among the different stages of development in the hen ovarian follicles. Of which, a highest level of *SAV1* mRNA expression was determined at the smallest follicles sampled (<1 mm in diameter), and the endogenous expression level of *SAV1* mRNA appears down-regulated from the smallest prehierarchical follicles to the largest preovulatory follicles (F2-F1) in the ovary. In previous studies, SAV1 has been found to act with Hpo/Mst to repress cell proliferation and to restrict tissue growth in vivo [29, 30, 44]. The localization of SAV1 protein and the dynamic changes at mRNA expression levels may suggest a close association between different regulative capability of SAV1 on follicular growth and the developmental stages corresponding to the various sized follicles.

It has been demonstrated that the core members of the Hippo pathway are in part coordinated through oocyte maturation, granular cell proliferation, follicular atresia, and other physiological processes within the mammalian ovary [10, 27, 46]. Wherein, SAV1 functions to promote the phosphorylation and activation of the large tumor suppressor kinase Lats1 by Ste-20 kinase Mst2 in mammals [30, 31], and Lats, Sav and Msts might form a trimeric complex in which Sav functions as an adaptor protein to bring Wts/Lats in close proximity to Hpo/Mst [47]. Moreover, a direct interaction was observed between Mst2 and hWW45/Sav in human [31]. Here, our data confirmed that chicken SAV1 is not only binding directly with STK4 and/or STK3, but also physically interacts with LATS1 and/or MOB2 in the GCs of the prehierarchical follicles in the ovary. This result suggested that the core members of the Hippo pathway, comprising at least one of the upstream Ste-20 kinases (STK4 and STK3), SAV1 and LATS1 as well as MOB2 molecule might form a larger and more complex functional compound in which SAV1 serves as a connector to assembly them together through a mode of action similar to the previously reported in mammals [47]. Furthermore, the SAV1 molecule was reported to have several phosphorylation residues that were phosphorylated by MST in human [33, 34, 35]. In this study, we found that chicken SAV1 was also phosphorylated by SKT4 or SKT3 kinase of the Hippo/MST pathway in the GCs of the developing ovarian follicles, and the phosphorylated SAV1 was able to significantly enhance the phosphorylation levels of LATS1 induced by the kinase of STK4 or STK3 in vitro. Studies have indicated that the phosphorylated LATS1 then phosphorylates and inactivates the downstream transcriptional co-activator YAP1 that is responsible for the expression of multiple apoptosis-related genes [18, 48, 49]. Therefore, the phosphorylation of chicken SAV1 mediated by the Ser/Thr protein kinase STK4 or STK3 may be most important in the regulation of oocyte maturation and granular cell proliferation during ovarian follicle growth by the Hippo/MST pathway. As for whether the detailed phosphorylated residues within the chicken SAV1 is consistent to those having been determined in mammals [35], further studies will be required to address this possibility.

To further explore the roles of SAV1 in development of chicken ovarian follicles, SAV1 knockdown and overexpression were performed in this study. We found that knockdown of SAV1 induced the significant increase in proliferation of the granular cells with the expression levels of *GDF9*, *StAR* and *FSHR* mRNA abundance (except for *CCND2* gene) were markedly enhanced. It have been previously reported that GDF9 and StAR factors exert a pivotal function in granulosa cell proliferation and differentiation, and in oocyte maturation as well as in the steroidogenesis during ovarian follicle growth [4, 6, 39, 50, 51], and then they act as a biomarker for the granulosa cell differentiation and proliferation [4, 7, 39, 50]. Furthermore, FSHR is required for follicular development and GC proliferation and differentiation by mediating stimulation of pituitary FSH [39]. In the hen ovary, selection of a dominant follicle into the preovulatory hierarchy occurs from a small cohort of prehierarchal (6–8 mm) follicles [8, 52]. Prior to follicle selection, a higher level of *FSHR* mRNA in GCs was required for the selection of PF 6–8 mm in diameter into the preovulatory hierarchy [8, 39, 52]. Herein, the enhancement of expression levels of *FSHR* mRNA in the GCs implied SAV1 was tightly correlated with the follicle selection. Thus, on the basis of our present data in addition to the previous reports, it was suggested that down-regulation of SAV1 may play an important role in governing the selection of dominant prehierarchical follicles into a preovulatory stage, as well as in promoting the oocyte maturation and granular cell proliferation during ovarian follicle development in chicken. On the other hand, in the current study, the data showed that the GC proliferation index was significantly reduced, in which the expression levels of *GDF9*, *StAR* and *FSHR* mRNA (including *CCND2* gene) were notably decreased by the SAV1 overexpression. Our present result of SAV1 up-regulating expression likewise consolidated that SAV1 plays a suppressive role in ovarian follicle development, and it may result in hindering the follicle selection mediated by the Hippo/MST pathway. The current data also confirmed that it is reliable to utilize the *GDF9* or *StAR* gene as a marker of granulosa cell differentiation and proliferation.

It has been reported that the cyclin D2 (CCND2) expression is localized to the granulosa cells of the ovarian follicles [53], FSH acutely increased granulosa cell *CCND2* mRNA abundance and CCND2 protein content as well as proliferation [54], and human granulosa cell tumors display high levels of *CCND2* mRNA compared with wild-type ovaries [53]. Conversely, the hypophysectomized female rats display low levels of cyclin D2 in the ovaries and are unable to sustain follicular growth or to stimulate granulosa cell proliferation [55]. Therefore, CCND2 is an important factor in the regulation of granulosa cell proliferation during ovarian folliculogenesis [54, 56, 57]. *CCND2* gene was considered as promising biomarker of granular cell differentiation [5, 7, 57]. However, in the current data, a divergent effect on expression of *CCND2* abundance in GCs was detected by the SAV1 overexpression and the SAV1 knockdown. Considering the exact roles and regulative mechanism of SAV1 in development of the ovary follicle is currently unclear, further study to clarify whether SAV1 factor has a dispensable effect on chicken *CCND2* gene in the ovary follicle is warranted.

## Conclusion

This study initially confirmed that the chicken SAV1, as a member of the hippo/MST signaling pathway which plays a suppressive role in ovarian follicle development by promoting phosphorylation and activity of the downstream LATS1 induced by the kinase of STK4 or STK3, may consequently lead to prevention of the follicle selection during ovary development.

## Supporting Information

S1 Table Antibodies and blocking peptides used for immunohistochememistry. (DOC)

S2 Table Antibodies used for Western blot analysis. (DOC)

## References

[1] Gilchrist RB, Ritter LJ, Myllymaa S, Kaivo-Oja N, Dragovic RA, Hickey TE, et al Molecular basis of oocyte-paracrine signaling that promotes granulosa cell proliferation. J Cell Sci. 2006; 119: 3811–3821. 16926195 10.1242/jcs.03105

[2] Woodruff TK, Shea LD. The Role of the extracellular matrix in ovarian follicle development. Reprod Sci. 2007; 14: 6–10. 10.1177/1933719107309818 PMC2648348 18089604

[3] Qin N, Fan XC, Zhang YY, Xu XX, Tyasi TL, Jing Y, Mu F, Wei ML, Xu RF. New insights into implication of the SLIT/ROBO pathway in the prehierarchical follicle development of hen ovary. Poult Sci. 2015; 94: 2235–2246. 10.3382/ps/pev185 26188027

[4] Dong J, Albertini DF, Nishimori K, Kumar TR, Lu N, Matzuk MM. Growth differentiation factor-9 is required during early ovarian folliculogenesis. Nature. 1996; 383: 531–535. 8849725 10.1038/383531a0

[5] Bentsi-Barnes IK, Kuo FT, Barlow GM, Pisarska MD. Human forkhead L2 represses key genes in granulosa cell differentiation including aromatase, P450scc, and cyclin D2. Fertil Steril. 2010; 94:353–356. 10.1016/j.fertnstert.2009.09.050 19917504 PMC2876195

[6] Lin D, Sugawara T, Strauss JF 3rd, Clark BJ, Stocco DM, Saenger P, Rogol A, Miller WL. Role of steroidogenic acute regulatory protein in adrenal and gonadal steroidogenesis. Science 1995; 267: 1828–1231. 7892608 10.1126/science.7892608

[7] Pisarska MD, Kuo FT, Bentsi-Barnes IK, Khan S, Barlow GM. LATS1 phosphorylates forkhead L2 and regulates its transcriptional activity. Am J Physiol Endocrinol Metab. 2010; 299(1): E101–9. 20407010 10.1152/ajpendo.00534.2009 PMC2904049

[8] Johnson AL. Ovarian follicle selection and granulosa cell differentiation. Poult Sci. 2015; 94: 781–785. 10.3382/ps/peu008 25535403

[9] Kawamura K, Cheng Y, Suzuki N, Deguchi M, Sato Y, Takae S, Ho CH, Kawamura N, Tamura M, Hashimoto S, Sugishita Y, Morimoto Y, Hosoi Y, Yoshioka N, Ishizuka B, Hsueh AJ. Hippo signaling disruption and Akt stimulation of ovarian follicles for infertility treatment. Proc Natl Acad Sci U S A. 2013; 110:17474–17479. 10.1073/pnas.1312830110 24082083 PMC3808580

[10] Xiang C, Li J, Hu L, Huang J, Luo T, Zhong Z, Zheng Y, Zheng L. Hippo signaling pathway reveals a spatio-temporal correlation with the size of primordial follicle pool in mice. Cell Physiol Biochem. 2015; 35: 957–968. 10.1159/000369752 25659841

[11] Edgar BA. From cell structure to transcription: Hippo forges a new path. Cell. 2006; 124: 267–273. 16439203 10.1016/j.cell.2006.01.005

[12] Harvey K, Tapon N.The Salvador-Warts-Hippo pathway—an emerging tumour-suppressor network. Nat Rev Cancer. 2007; 7: 182–191. 17318211 10.1038/nrc2070

[13] Zhao B, Lei QY, Guan KL: The hippo-yap pathway: New connections between regulation of organ size and cancer. Curr Opin Cell Biol. 2008; 20: 638–46. 10.1016/j.ceb.2008.10.001 18955139 PMC3296452

[14] Kodaka M, Hata Y. The mammalian Hippo pathway: regulation and function of YAP1 and TAZ. Cell Mol Life Sci. 2015; 72: 285–306. 10.1007/s00018-014-1742-9 25266986 PMC11113917

[15] Lange AW, Sridharan A, Xu Y, Stripp BR, Perl AK, Whitsett JA. Hippo/Yap signaling controls epithelial progenitor cell proliferation and differentiation in the embryonic and adult lung. J Mol Cell Biol. 2015; 7: 35–47. 10.1093/jmcb/mju046 25480985 PMC4400400

[16] Hergovich A, Stegert MR, Schmitz D, Hemmings BA. NDR kinases regulate essential cell processes from yeast to humans. Nat Rev Mol Cell Biol. 2006; 7: 253–264. 16607288 10.1038/nrm1891

[17] Hergovich A, Schmitz D, Hemmings BA. The human tumour suppressor LATS1 is activated by human MOB1 at the membrane. Biochem Biophys Res Commun. 2006; 345: 50–58. 16674920 10.1016/j.bbrc.2006.03.244

[18] Zhao B, Ye X, Yu J, Li L, Li W, Li S, Yu J, Lin JD, Wang CY, Chinnaiyan AM, Lai ZC, Guan KL.TEAD mediates YAP-dependent gene induction and growth control. Genes Dev. 2008; 22: 1962–1971. 10.1101/gad.1664408 18579750 PMC2492741

[19] Pan D. The hippo signaling pathway in development and cancer. Dev Cell. 2010; 19: 491–505. 10.1016/j.devcel.2010.09.011 20951342 PMC3124840

[20] Halder G, Johnson RL. Hippo signaling: Growth control and beyond. Development. 2011; 138: 9–22. 10.1242/dev.045500 21138973 PMC2998162

[21] Zhou D, Zhang Y, Wu H, Barry E, Yin Y, Lawrence E, Dawson D, Willis JE, Markowitz SD, Camargo FD, Avruch J. Mst1 and Mst2 protein kinases restrain intestinal stem cell proliferation and colonic tumorigenesis by inhibition of Yes-associated protein (Yap) overabundance. Proc Natl Acad Sci U S A. 2011; 108: E1312–20. 10.1073/pnas.1110428108 22042863 PMC3241824

[22] Karpowicz P, Perez J, Perrimon N. The Hippo tumor suppressor pathway regulates intestinal stem cell regeneration. Development 2010; 137:4135–4145. 10.1242/dev.060483 21098564 PMC2990205

[23] Barry ER, Camargo FD. The Hippo superhighway: signaling crossroads converging on the Hippo/Yap pathway in stem cells and development. Curr Opin Cell Biol. 2013; 25:247–253. 10.1016/j.ceb.2012.12.006 23312716

[24] Hao J, Zhang Y, Jing D, Li Y, Li J, Zhao Z: Role of Hippo signaling in cancer stem cells. J Cell Physiol. 2014; 229: 266–270. 10.1002/jcp.24455 24037831

[25] Lu L, Li Y, Kim SM, Bossuyt W, Liu P, Qiu Q, Wang Y, Halder G, Finegold MJ, Lee JS, Johnson RL. Hippo signaling is a potent in vivo growth and tumor suppressor pathway in the mammalian liver. Proc Natl Acad Sci U S A. 2010; 107: 1437–1442. 10.1073/pnas.0911427107 20080689 PMC2824398

[26] Sarikaya DP, Extavour CG. The Hippo pathway regulates homeostatic growth of stem cell niche precursors in the Drosophila ovary. PLoS Genet. 2015; 11(2): e1004962 10.1371/journal.pgen.1004962 25643260 PMC4333732

[27] Cheng Y, Feng Y, Jansson L, Sato Y, Deguchi M, Kawamura K, Hsueh AJ. Actin polymerization-enhancing drugs promote ovarian follicle growth mediated by the Hippo signaling effector YAP. FASEB J. 2015; 29: 2423–2430. 10.1096/fj.14-267856 25690654 PMC4447223

[28] Tapon N, Harvey KF, Bell DW, Wahrer DC, Schiripo TA, Haber D, Hariharan IK. salvador Promotes both cell cycle exit and apoptosis in Drosophila and is mutated in human cancer cell lines. Cell 2002; 110: 467–78. 12202036 10.1016/s0092-8674(02)00824-3

[29] Matsuura K, Nakada C, Mashio M, Narimatsu T, Yoshimoto T, Tanigawa M, Tsukamoto Y, Hijiya N, Takeuchi I, Nomura T, Sato F, Mimata H, Seto M, Moriyama M. Downregulation of SAV1 plays a role in pathogenesis of high-grade clear cell renal cell carcinoma. BMC Cancer. 2011; 11:523 10.1186/1471-2407-11-523 22185343 PMC3292516

[30] Wu S, Huang J, Dong J, Pan D. hippo encodes a Ste-20 family protein kinase that restricts cell proliferation and promotes apoptosis in conjunction with salvador and warts. Cell 2003; 114: 445–456. 12941273 10.1016/s0092-8674(03)00549-x

[31] Chan EH, Nousiainen M, Chalamalasetty RB, Schäfer A, Nigg EA, Silljé HH. The Ste20-like kinase Mst2 activates the human large tumor suppressor kinase Lats1. Oncogene 2005; 24: 2076–2086. 15688006 10.1038/sj.onc.1208445

[32] Lee JH, Kim TS, Yang TH, Koo BK, Oh SP, Lee KP, Oh HJ, Lee SH, Kong YY, Kim JM, Lim DS. A crucial role of WW45 in developing epithelial tissues in the mouse. EMBO J. 2008; 27: 1231–1242. 10.1038/emboj.2008.63 18369314 PMC2367404

[33] Hwang E, Ryu KS, Pääkkönen K, Güntert P, Cheong HK, Lim DS, Lee JO, Jeon YH, Cheong C. Structural insight into dimeric interaction of the SARAH domains from Mst1 and RASSF family proteins in the apoptosis pathway. Proc Natl Acad Sci U S A. 2007; 104: 9236–9241. 17517604 10.1073/pnas.0610716104 PMC1890478

[34] Callus BA, Verhagen AM, Vaux DL. Association of mammalian sterile twenty kinases, Mst1 and Mst2, with hSalvador via C-terminal coiled-coil domains, leads to its stabilization and phosphorylation. FEBS J. 2006; 273: 4264–4276. 16930133 10.1111/j.1742-4658.2006.05427.x

[35] Park BH, Lee YH. Phosphorylation of SAV1 by mammalian ste20-like kinase promotes cell death. BMB Rep. 2011; 44: 584–589. 21944251 10.5483/bmbrep.2011.44.9.584

[36] Qin N, Liu Q, Zhang YY, Fan XC, Xu XX, Lv ZC, Wei ML, Jing Y, Mu F, Xu RF. Association of novel polymorphisms of forkhead box L2 and growth differentiation factor-9 genes with egg production traits in local Chinese Dagu hens. Poult Sci. 2015, 94: 88–95. 10.3382/ps/peu023 25577797

[37] Stepińska U, Olszańska B. Characteristics of poly (A)-degrading factor present in the avian oocytes and early embryos. J Exp Zool. 1996; 276: 19–29. 8828183 10.1002/(SICI)1097-010X(19960901)276:1<19::AID-JEZ3>3.0.CO;2-8

[38] Johnson PA, Dickens MJ, Kent TR, Giles JR. Expression and function of growth differentiation factor-9 in an oviparous species. Gallus domesticus. Biol Reprod. 2005; 72: 1095–1100. 15625233 10.1095/biolreprod.104.036822

[39] Qin N, Fan XC, Xu XX, Tyasi TL, Li SJ, Zhang YY, Wei ML, Xu RF. Cooperative Effects of FOXL2 with the members of TGF-β superfamily on FSH receptor mRNA expression and granulosa cell proliferation from hen prehierarchical follicles. PLoS One. 2015; 10 (10): e0141062 10.1371/journal.pone.0141062 26496659 PMC4619702

[40] Lun YZ, Chi Q, Wang XL, Wang F, Sui W. Identification of paired immunoglobulin-like type 2 receptor α as hepatitis B virus DNA polymerase transactivated protein 1 interacting proteins. Mol Med Rep. 2014; 9:720–724. 10.3892/mmr.2013.1813 24253495

[41] Kuo FT, Bentsi-Barnes IK, Barlow GM, Bae J, Pisarska MD. Sumoylation of forkhead L2 by Ubc9 is required for its activity as a transcriptional repressor of the Steroidogenic Acute Regulatory gene. Cell Signal. 2009; 21: 1935–1944. 10.1016/j.cellsig.2009.09.001 19744555 PMC2830813

[42] http://www.sirnawizard.com/design_advanced.php, accessed on February 6th, 2015.

[43] Lu WD, Zhu YL, Sha J, Zhu HB. SPSS for Windows Statistical Analysis (2nd ed). Beijing: Publishing House of Electronics Industry; 2002 pp.124–143 (in Chinese).

[44] Pantalacci S, Tapon N, Léopold P. The Salvador partner Hippo promotes apoptosis and cell-cycle exit in Drosophila. Nat Cell Biol. 2003; 5: 921–927. 14502295 10.1038/ncb1051

[45] Xiang C, Li J, Hu L, Huang J, Luo T, Zhong Z, Zheng Y, Zheng L. Hippo signaling pathway reveals a spatio-temporal correlation with the size of primordial follicle pool in mice. Cell Physiol Biochem. 2015; 35: 957–968. 10.1159/000369752 25659841

[46] Li J, Zhou F, Zheng T, Pan Z, Liang X, Huang J, Zheng L, Zheng Y. Ovarian germline stem cells (OGSCs) and the hippo signaling pathway association with physiological and pathological ovarian aging in mice. Cell Physiol Biochem. 2015; 36:1712–1724. 26183517 10.1159/000430144

[47] Harvey KF, Pfleger CM, Hariharan IK. The Drosophila Mst ortholog, hippo, restricts growth and cell proliferation and promotes apoptosis. Cell. 2003; 114: 457–467. 12941274 10.1016/s0092-8674(03)00557-9

[48] Li T, Zhao H, Zhao X, Zhang B, Cui L, Shi Y, Li G, Wang P, Chen ZJ. Identification of YAP1 as a novel susceptibility gene for polycystic ovary syndrome. J Med Genet. 2012; 49: 254–257. 10.1136/jmedgenet-2011-100727 22499345

[49] Zhao B, Wei X, Li W, Udan RS, Yang Q, Kim J, Xie J, Ikenoue T, Yu J, Li L, Zheng P, Ye K, Chinnaiyan A, Halder G, Lai ZC, Guan KL. Inactivation of YAP oncoprotein by the Hippo pathway is involved in cell contact inhibition and tissue growth control. Genes Dev. 2007; 21: 2747–2761. 17974916 10.1101/gad.1602907 PMC2045129

[50] Hayashi M, McGee EA, Min G, Klein C, Rose UM, van Duin M, et al Recombinant growth differentiation factor-9 (GDF-9) enhances growth and differentiation of cultured early ovarian follicles. Endocrinology. 1999; 140: 1236–1244. 10067849 10.1210/endo.140.3.6548

[51] Thompson WE, Powell J, Thomas KH, Whittaker JA. Immunolocalization and expression of the steroidogenic acute regulatory protein during the transitional stages of rat follicular differentiation. J Histochem Cytochem. 1999; 47: 769–776. 10330453 10.1177/002215549904700606

[52] Woods DC, Johnson AL. Regulation of Follicle-Stimulating Hormone receptor mRNA in hen granulosa cells relative to follicle selection. Biol Reprod. 2005; 72: 643–650. 15537865 10.1095/biolreprod.104.033902

[53] Sicinski P, Donaher JL, Geng Y, Parker SB, Gardner H, Park MY, Robker RL, Richards JS, McGinnis LK, Biggers JD, Eppig JJ, Bronson RT, et al Cyclin D2 is an FSH-responsive gene involved in gonadal cell proliferation and oncogenesis. Nature 1996; 384: 470–474 8945475 10.1038/384470a0

[54] Han Y, Xia G, Tsang BK. Regulation of cyclin D2 expression and degradation by follicle-stimulating hormone during rat granulosa cell proliferation in vitro. Biol Reprod. 2013; 88 (3): 57 10.1095/biolreprod.112.105106 23349233

[55] Robker RL, Richards JS, Hormone-induced proliferation and differentiation of granulosa cells: a coordinated balance of the cell cycle regulators cyclin D2 and p27Kip1. Mol Endocrinol 1998; 12: 924–940. 9658398 10.1210/mend.12.7.0138

[56] Muñiz LC, Yehia G, Mémin E, Ratnakar PV, Molina CA. Transcriptional regulation of cyclin D2 by the PKA pathway and inducible cAMP early repressor in granulosa cells. Biol Reprod. 2006; 75: 279–288. 16625003 10.1095/biolreprod.105.049486

[57] Kuo FT, Fan K, Bentsi-Barnes I, Barlow GM, Pisarska MD. Mouse forkhead L2 maintains repression of FSH-dependent genes in the granulosa cell. Reproduction 2012; 144: 485–494. 10.1530/REP-11-0259 22847492

