# Supplementary material for: The Hippo/MST Pathway Member SAV1 Plays a Suppressive Role in Development of the Prehierarchical Follicles in Hen Ovary
Source: PLoS One. 2016 Aug 9;11(8):e0160896. doi: 10.1371/journal.pone.0160896 (PMC4978403; doi:10.1371/journal.pone.0160896)
Supplement: S1 Table — (DOC) [file pone.0160896.s001.doc]

**S1 Table Primer pairs used for reconstruction of the expression vectors.**

| Gene | Recombinant vector | Primer pairs (the forward and reverse, 5′ - 3′) | Restriction sites |
| --- | --- | --- | --- |
| *SAV1* | pFLAG-SAV1 | ATTTGCGGCCGCG**ATG**CTGTCGCGGAAGAAAAC  ACGCGGATCCtcaGAAGTTCTTGCCGTGCTGCTG | Not I  BamH I |
| *SAV1* | pSF-GST-SAV1 | CGGAATTCT**ATG**CTGTCGCGGAAGAAAAC  ATATGGATCCCTtcaGAAGTTCTTGCCGTGCTGCTG | EcoR I  BamH I |
| *STK4* | pcDNA3.0-STK4 | ATTTGCGGCCGCA**ATG**GAGACGGTGCAGCTGCGG  GCCTCGAGTtcaGAAGTTCTGCTGCCGCCGC | Not I  Xho I |
| *STK3* | pcDNA3.0-STK3 | CGGGTACCGGA**ATG**GAGCAGGCAGCGCCCAAG  GCGGATCCtcaGAAATTTTGTTGTCTCCTC | Kpn I  BamH I |
| *LATS1* | pcDNA3.0-LATS1 | CGGGTACCGGA**ATG**AAGAGAAGTGAGAAGCC  GCGCGGCCGCTctaAACATAAACTAGGTCACG | Kpn I  Not I |
| *LATS1* | pCMV-HA-LATS1 | CGGTCGACT**ATG**AAGAGAAGTGAGAAGCC  ATATGGTACCTctaAACATAAACTAGGTCACG | Sal I  Kpn I |
| *MOB2* | pcDNA3.0-MOB2 | CGAAGCTTGGA**ATG**GTCGGGGATCAGTGCAGC  GCGGATCCtcaTCTCTCTTTCACGTGGTTC | Hind Ⅲ  BamH I |

Note: The underlined nucleotides indicated the location corresponding to each of the restriction sites.
