# Supplementary material for: The Hippo/MST Pathway Member SAV1 Plays a Suppressive Role in Development of the Prehierarchical Follicles in Hen Ovary
Source: PLoS One. 2016 Aug 9;11(8):e0160896. doi: 10.1371/journal.pone.0160896 (PMC4978403; doi:10.1371/journal.pone.0160896)
Supplement: S2 Table — (DOC) [file pone.0160896.s002.doc]

**S2 Table. Antibodies used for Western blot analysis**

| **Protein target** | **Primary**  **antibody** | **Dilution used** | **Antibody type** | **Secondary**  **antibody** | **Dilution used** | **Originated** |
| --- | --- | --- | --- | --- | --- | --- |
| SAV1 | Rabbit anti- SAV1 | 1/1000 | Monoclonal | anti-rabbit IgG | 1/2000 | Sangon Co, Shanghai, China |
| STK4 | Mouse anti- STK4 | 1/2000 | polyclonal | anti- mouse IgG | 1/5000 | Invitrogen, Carlsbad, CA, USA |
| SIK3 | Mouse anti- STK3 | 1/2000 | polyclonal | anti-mouse IgG | 1/5000 | Invitrogen, Carlsbad, CA, USA |
| LATS1 | Rabbit anti- LATS1 | 1/1000 | Monoclonal | anti- rabbit IgG | 1/3000 | Sangon Co, Shanghai, China |
| MOB2 | Rabbit anti- MOB2 | 1/500 | polyclonal | anti-rabbit IgG | 1/2000 | Rockford, IL, USA |
| YAP1 | Rabbit anti- YAP1 | 1/500 | Monoclonal | anti-rabbit IgG | 1/2000 | Sangon Co, Shanghai, China |
| pSAV1 | Rabbit anti- pLATS1 | 1/1000 | Monoclonal | anti-rabbit IgG | 1/3000 | Sangon Co, Shanghai, China |
| pLATS1 | Mouse anti- pLATS1 | 1/1000 | Monoclonal | anti-mouse IgG | 1/5000 | Sangon Co, Shanghai, China A |
| β-actin | Mouse anti- β-actin | 1/1000 | polyclonal | anti-mouse IgG | 1/2000 | Boster Biological Technology, China |

Note: Source of the antibodies against the chicken protein/peptide target list in this table. pSAV1: chicken phospho- SAV1, pLATS1: chicken phospho- LATS1. Antibody concentration: 1μg/ul, size: 100 ul.
